# THE AGING RESEARCH IN CRIMINAL JUSTICE HEALTH (ARCH) NETWORK: AN OVER-ARCH-ING VIEW

**DOI:** 10.1093/geroni/igae098.1545

**Published:** 2024-12-31

**Authors:** Lisa Barry, Nickolas Zaller, Brie Williams

**Affiliations:** UConn Center on Aging, Farmington, Connecticut, United States; University of Arkansas for medical sciences, Little Rock, Arkansas, United States; University of California San Francisco, San Francisco, California, United States

## Abstract

Criminal justice-involved older adults have high rates of early onset disease and disability and they experience significant health disparities. Yet, research focused on this population and the consequences of criminal justice involvement on health in later life is deeply under-developed. In response, the Aging Research in Criminal Justice Health Network was established in 2019 and we are excited to celebrate its achievements! We will discuss the process of building this network and how the ARCH Network has made significant strides in promoting the importance of considering geriatric conditions (e.g., functional and cognitive impairment, falls, polypharmacy) when evaluating the health of justice-involved older adults. We will also highlight accomplishments including the significant number of publications championed by the ARCH Network and the successful creation of a GSA Interest Group devoted to Incarceration and Aging.

